# A Case Study on the Integration of Powerline Communications and Visible Light Communications from a Power Electronics Perspective

**DOI:** 10.3390/s24206627

**Published:** 2024-10-14

**Authors:** Felipe Loose, Juan Ramón Garcia-Meré, Adrion Andrei Rosanelli, Carlos Henrique Barriquello, José Antonio Fernandez Alvárez, Juan Rodríguez, Diego González Lamar

**Affiliations:** 1 Department of Electrical Engineering, Electronics, Communications and Systems, University of Oviedo (UNIOVI), 33204 Gijón, Spain; garciamjuan@uniovi.es (J.R.G.-M.); fernandezalvantonio@uniovi.es (J.A.F.A.); rodriguezmjuan@uniovi.es (J.R.); gonzalezdiego@uniovi.es (D.G.L.); 2 Technology Center, Federal University of Santa Maria (UFSM), Santa Maria 97105-900, Brazil; aarosanelli@gmail.com (A.A.R.); barriquello@gedre.ufsm.br (C.H.B.)

**Keywords:** visible light communication, wireless communication, sensor networks, powerline communications

## Abstract

This paper presents a dual-purpose LED driver system that functions as both a lighting source and a Visible Light Communication (VLC) transmitter integrated with a Powerline Communication (PLC) network under the PRIME G3 standard. The system decodes PLC messages from the powerline grid and transmits the information via LED light to an optical receiver under a binary phase shift keying (BPSK) modulation. The load design targets a light flux of 800 lumens, suitable for LED light bulb applications up to 10 watts, ensuring practicality and energy efficiency. The Universal Asynchronous Receiver-Transmitter (UART) module enables communication between the PLC and VLC systems, allowing for an LED driver with dynamic control and real-time operation. Key signal processing stages are commented and developed, including a hybrid buck converter with modulation capabilities and a nonlinear optical receiver to regenerate the BPSK reference signal for VLC. Results show a successful prototype working under a laboratory environment. Experimental validation shows successful transmission of bit streams from the PLC grid to the VLC setup. A design guideline is presented in order to dictate the design of the electronic devices involved in the experiment. Finally, this research highlights the feasibility of integrating PLC and VLC technologies, offering an efficient and cost-effective solution for data transmission over existing infrastructure.

## 1. Introduction

Visible Light Communications (VLC) and Powerline Communications (PLC) are two innovative technologies at the forefront of modern communication systems, each leveraging distinct mediums to transmit data seamlessly. VLC harnesses the spectrum of visible light, utilizing light-emitting diodes (LEDs) to transmit data, offering advantages as a wireless medium with intrinsic interesting features and free from radio-frequency interference [1,2,3]. PLC utilizes existing electrical wiring infrastructure to transmit data, facilitating communication through power lines, thereby enabling applications such as home automation and smart grid management [4,5]. Therefore, it is interesting to elaborate scenarios where these two forms of communication interact with each other in order to harvest the potentials of this unification.

Despite their differences in transmission mediums, both VLC and PLC share the common goal of enhancing connectivity and efficiency in various domains, driving the evolution of communication technologies towards a more interconnected and data-driven future. However, integrating VLC and PLC into existing communication networks poses significant challenges. Hence, overcoming these limitations requires innovative signal processing techniques, sophisticated electronics, and adaptive algorithms to optimize performance and reliability.

Moreover, the need to ensure a reliable, robust, and easy-to-deploy communication system is a mandatory subject in order to reduce costs and make the integration feasible for mediums targeting low to moderate data rate applications. Such applications may involve sensor networks, smart grid monitoring, and control and intelligent indoor position systems for human and robot localization.

In this work, we explore some of the popular forms of integration between PLC and VLC. We navigate through three possible applications scenarios and two integration possibilities, commonly called Analog and Forward (AF) and Digitalize and Forward (DF) integrations [6]. We address potential challenges while offering a case study on a prototype LED lamp based on the DF solution. We approach our subject mainly through the lenses of power electronics and communications, analyzing potential challenges for integrating this technology considering the electronics behind it and its industrial acceptance.

Hence, the integrated prototype exemplifies a PLC-VLC system, offering a comprehensive solution for seamless data transmission within indoor environments. We provide a proof of concept within the scope of technology readiness level 4 (TRL 4) [7].

## 2. Literature Review

Popular applications of PLC are widespread in power processing-oriented devices. These devices include smart meters, home automation systems, energy management systems, and industrial control systems. PLC facilitates real-time monitoring, control, and management of power consumption, allowing for efficient energy distribution and optimization.

Hence, because of its more matured level, we address the PLC-VLC integration through the sense of applying VLC into possible PLC scenarios in order to enhance their potential in terms of user experience and connectivity. Next, we address some interesting advantages VLC may offer to already existent PLC applications [2,8].

- High Security: VLC offers inherent security advantages due to its directional nature and the inability of light to penetrate opaque obstacles and also walls. Integrating VLC into PLC networks enhances security by reducing the risk of eavesdropping or interception compared to radio-frequency (RF) communication technologies. This is particularly beneficial in applications requiring secure data transmission, such as smart grid systems and industrial automation.
- Infrastructure Reuse: VLC-enabled light fixtures can serve dual purposes by providing illumination and communication simultaneously, without the need of deploying extra hardware for the communication network.
- Low Interference and Electromagnetic Compatibility: VLC operates in the optical spectrum, which is immune to electromagnetic interference (EMI) and radio-frequency interference (RFI) that often affect PLC signals. Integrating VLC into PLC networks mitigates the effects of interference, ensuring reliable communication even in environments with high levels of electrical, such as industrial scenarios of application.

Furthermore, we also address some popular applications that we believe would be favored by VLC.

### 2.1. Smart Meters

Smart meters are integral components of advanced metering infrastructure (AMI) used for measuring and monitoring electricity consumption in residential, commercial, and industrial settings. Smart meters transmit consumption data, status updates, and diagnostic information to utility companies for billing, grid management, and load balancing purposes. The data rates typically range from moderate to high, considering the volume and granularity of data transmitted. For example, basic devices may transmit data periodically at every 15 min, with small packets and at moderate data rates from 100 to 500 kpbs, with bandwidths also from 100 to 500 kHz [9].

We believe this type of application scenario to be worth exploring into PLC-VLC solutions. The data rates and hardware requirements are already consolidated, and VLC would not demand a severe retrofit design in terms of hardware components, as will be shown next on the prototype section. Here, VLC operates as a front end wireless communication system, offering a reliable and fast approach to visualize and gather data processed by smart devices into a receiver working as a mobile data storage system, such as a smartphone or a tablet.

### 2.2. Home Automation Systems

Home automation systems encompass a wide range of devices and applications, including lighting control; heating, ventilation, and air conditioning (HVAC) systems; security systems; and entertainment systems [10].

The data rate in home automation systems can vary widely depending on factors such as the number of devices, the frequency of command and status updates, the type of data being transmitted (e.g., sensor readings, control commands), and the responsiveness required from the system. For example, real-time control commands may necessitate higher data rates compared to periodic status updates.

Furthermore, aiming at a possible industry-friendly integration between PLC and VLC, we believe the targets shall be on devices that would offer a more profitable margin and scalability, while also exploring the same features encountered in the integration of smart metering devices. Thus, VLC not only offers a wireless medium that encloses the transfer of data into one single room, due to its intrinsic features concerning the scatter of light, but may also work as a broadcast service communicating with several devices at once.

### 2.3. Indoor Positioning

Indoor positioning consists of technologies able to localize end users inside dense infrastructures. Similar approaches have been revisiting light-based technologies in the infrared spectrum and visible light as a complementary wireless medium to perform such task [11,12].

With VLC in indoor positioning, light is used as the communication medium in a diffuse way to enable communication between smart devices scattered throughout a room illuminated by artificial light via smart VLC lamps. For example, mobile devices, drones, and smart vehicles are machines that can leverage the potential of indoor positioning enabled by VLC by applying a ready-to-use communication medium through visible light [13].

The integration offers the potential of utilizing the already mounted wired infrastructure of the lighting system to deploy smart lamps with full duplex communication between each other via PLC and a reliable wireless broadcast service through VLC. In other words, PLC is used to form a network between the agents (the lamps) responsible for delivering the final positioning coordinates to photo-sensitive receivers. Such feature opens the door for direct synchronization of the network through PLC, enhancing positioning features and offering more precision in the service. Finally, VLC is used again as the front end application to easily deliver the information to the final user.

Therefore, these three highlighted applications form the basis to justify the integration for VLC in the PLC technology. Here, VLC offers a low cost and reliable wireless solution, specially enhancing the user access and experience. By working with low to moderate data rates being transmitted through light in a diffuse way, VLC offers easy user access to information being processed by PLC networks, working as a friendly wireless front-end for the application examples aforementioned.

### 2.4. Bandwidth and Commercial Applications

One important aspect in the integration part relies on the available bandwidth and modulations for PLC. In this sense, the market is already well-oriented and standardized, offering a series of solutions to perform the task. The available bandwidth applications can vary depending on various factors, such as the specific standard used, the conditions of the power network, and the technical limitations of the PLC devices. Below is an overview of typical bandwidths associated with different PLC standards and their protocols.

HomePlug: The latest versions of HomePlug standards, such as HomePlug AV2, are capable of offering speeds of up to several hundred megabits per second (Mbps) and, in some cases, speeds exceeding one gigabit per second (Gbps).

G.hn: G.hn is another standard that can support high data transmission speeds. It can provide bandwidth ranging from several hundred Mbps to Gbps, depending on the specific implementation and network conditions.

PRIME (ITU-T G.9904): This standard, designed for smart metering applications, can offer speeds ranging from tens to several hundred kilobits per second (kbps). Although not aimed at extremely high speeds, it is suitable for specific applications such as telemetry in power distribution systems.

IEEE P1901: The IEEE P1901 standard addresses various modulations and transmission speeds, meaning that the bandwidth can vary depending on the specific implementation. It can provide speeds ranging from several Mbps to Gbps [14].

G3: The G3 Power Line Communication (PLC) standard is designed specifically for smart grid applications. Its main focus is the ability to operate under noisy environments and also to facilitate scalability, both features highly demanded in industrial scenarios of power management.

In addition, concerning G3 and PRIME protocols, such solutions count with consolidated and reliable integrated circuits, working as PLC modems with the stack protocol already built in the integrated silicon chip. These solutions rely on an external microcontroller, apart from the modem device, as the manager of information, controlling the PLC modem via serial peripheral interface communications. Hence, this particular approach may be leveraged in order to provide a more oriented solution for the PLC-VLC integration by employing a commercially available PLC solution with consolidated quality while also favoring the approach of building a VLC structure as an additional feature stacked upon a PLC protocol and an already existent digital processor.

## 3. System Contextualization

The objective of this paper is to provide a concise description of an integrated PLC-VLC apparatus contained within the level 4 of the Technology Readiness Level (TRL) scale. Thus, with this approach, we seek to describe the potential challenges of such integration, and also provide the means to develop a reliable electronic LED driver capable of redirecting any information processed by a PLC channel through the modulated light of an LED load.

**Figure 1:**
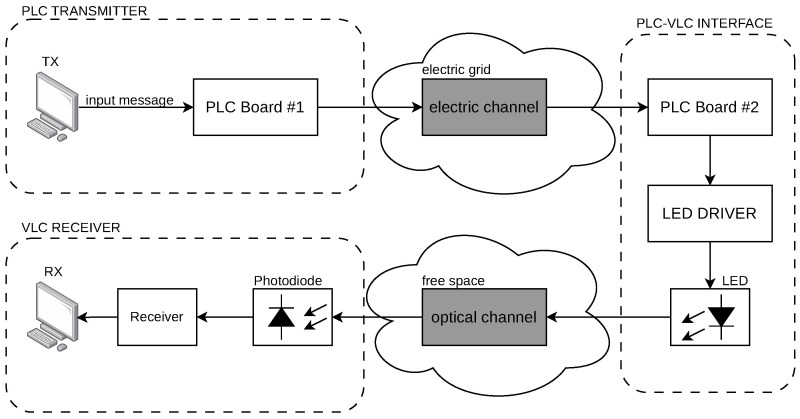
Block diagram of the system’s architecture.

Moreover, Figure 1 illustrates a block diagram of the proposed system. A message signal is generated by a Transmitter (TX) PC. The message travels to the electric grid (MAINS) through a PLC pair to be delivered to an LED driver for processing. The driver translates the message into electric current, forwarding it to an LED, thus enabling the electric-to-light conversion of the device. Finally, the message travels to the Free Space Optical Channel (FSOC) up to a photodiode receiver to be processed by a receiver module and finally delivered to a receiver (RX) PC. Hence, this setup shows a simplified version of a PLC-VLC scenario that contains all the challenges that will be addressed by this work and considering the LED driver design and the methods for forwarding the information between the two communication systems.

To finish this conceptualization, the techniques behind the PLC-VLC integration involve the choice of a given modulation scheme that can be processed by the digital unit of PLC Board #2, thus adapting modulations from PLC to a suited scheme for VLC. The advantage of operating this particular scheme in a digital form is the capacity of using distinct techniques for both applications, thus integrating them with the assistance of digital signal processing and computing power in a friendly-to-use system platform.

### 3.1. Challenges in the Integration of PLC-VLC

For a PLC-VLC system, the transmitted information must share two communication media of different physical origins. That is, the electrical medium, based on the transmission of electrical energy through cables where PLC technology is located, and the optical medium, accessed under the control of the electrical current of the LED load.

However, integrating a PLC-VLC system faces various technically challenging aspects. To exemplify, it is desirable to have an LED lamp capable of delivering the following capabilities: (i) providing electrical power to ensure ambient lighting; (ii) guaranteeing VLC capabilities through the modulation of LED currents; and (iii) providing a modulator system for PLC messages to process signals sent from the power grid. Ultimately, an electronic circuit capable of offering these three characteristics establishes the objective of this proof of concept.

#### 3.1.1. Integration

The challenges imposed by the idea of the lamp prototype and its aforementioned characteristics are highlighted. Designing a prototype of this nature faces two main challenges: (i) the need to obtain a manager interface for PLC messages to send them to the VLC interface and convert them into a current signal for the LED load; (ii) the need to supply electrical power to execute the lighting functions of the environment and to process the information signals for VLC.

For example, a prototype of this nature may be based on connecting a PLC circuit capable of processing information sent by the power grid and delivering it to a VLC lamp. According to the specialized literature, there are two methods to perform this transmission [6,15]:

1. An analog method, adopted by the acronym AF (Amplify and Forward), where the aim is to transmit the PLC signal directly from the power grid to the LED load current.
2. Another method based on the digitization of the electrical signal containing the PLC information, adopted by the acronym DF (Digitalize and Forward), for subsequent processing by the VLC circuit.

**Figure 2:**
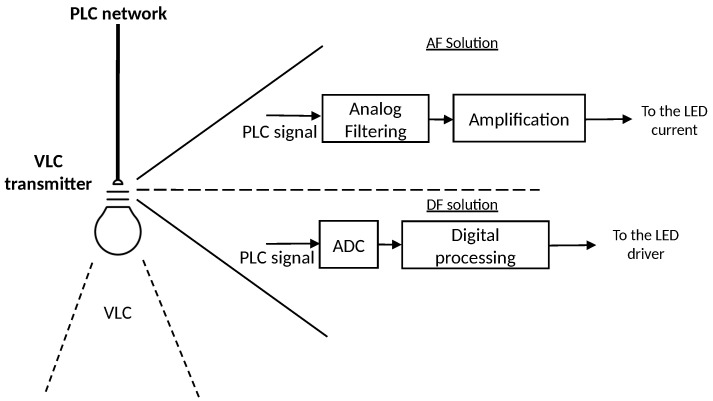
Differences between AF and DF solutions.

In addition, Figure 2 shows two simplified block diagrams containing the intrinsic features of each solution. To summarize, inside the VLC transmitter, an AF solution would require a set of passive and active electronic devices in order to successfully exactly match the PLC waveform into the LED current. On the other hand, a DF solution would be pass through and analog-to-digital converter (ADC), where a digital processing can be performed on the signal to be forwarded to the LED driver.

The difference lies in how the signal is processed in the two alternatives. The AF alternative directly sends the PLC signal to the LED current under analog processing. In this process, we address two main problems of the AF solution.

First, the feedback loop of the PLC channel is lost. PLC is a technology that relies on acknowledgment (ACK) signaling, and all protocols are devised to work on this confirmation message. However, in a PLC-VLC scenario, the signal is redirecting its message to the VLC transmitter and the message will only be demodulated by the end optical receiver that does not share any connection with the PLC loop, according to our contextualization. Thus, most PLC protocols would not successfully work in a direct forwarding of the PLC analog signal to the VLC system.

Second, dealing only with filtering and amplification imposes limitations in noise treatment of the integration. The impulsive noise, present in PLC and well-studied in the literature [16], will be merged to our sources of noise in the VLC domain, such as shot noise, which require more sophisticated tools that are now embedded into digital demodulation solutions of the PLC, through the integrated PLC modem chips [17].

Therefore, the DF solutions presents itself as a more well-developed candidate to operate the merging of these two communication systems. As will be seen in the next sections, the DF solutions also simplifies the connection between several digital agents that participate in the delivery of the message, as currently most digital systems are already built based on a similar interface.

#### 3.1.2. Channel Modeling

Another challenge lies on the treatment of noise present between the two media. In communications, scientists have been modeling how the environment interacts with the information signal that is traveling from one point to another. This interaction is usually described in the form of channel models that relate how the noise affects the message sent. Thus, like other communication systems, a PLC-VLC technology must be robust of noise to a certain level in order to allow the flow of information under desired levels of data rate.

In this context, specially concerning the VLC solution, the noise will be carried by the PLC channel and will incorporate the delivery of the message via the optical channel. Therefore, proper comments on the modeling of such noises may be addressed by this proof of concept.

**Figure 3:**
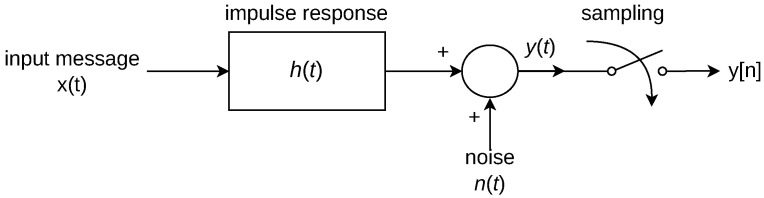
General channel model.

The general model employed is described as a discrete equivalent channel model as seen in Figure 3.

The input message is convoluted with the channel’s impulse response in which its result is affected by additive noise. Sampling may be applied to the resulting signal y(t) if a digital treatment is desired at the output, such as in the DF solution. Overall, the channel in the PLC-VLC system will differ in terms of the shape of the impulse response and the noise model employed to each solution.

#### 3.1.3. PLC Channel Model

The PLC channel is predominantly subject to background noise and impulsive noise (IN). The IN is not permanent and can be modeled by a Poisson distribution, so that at the output of the discrete channel a noise sample is given by [6,18]:

(1) $$n_{\mathrm{PLC}}=n_{G}+n_{I}\sqrt{D}$$

where $n_{G}$ and $n_{I}$ are, respectively, the background noise and the IN samples, following normal distributions with zero mean and variances $\sigma_{G}^{2}$ and $\sigma_{I}^{2}$. *D* is the Poisson distributed sequence whose probability density functions is characterized by the impulsive index *A*.

Therefore, the discrete noisy PLC channel output *y*, for a transmitted symbol *x*, is given by:

(2) $$y=hx+n_{\mathrm{PLC}},$$

where *h* is the PLC channel gain, corresponding to a constant gain for the channel impulse response.

#### 3.1.4. VLC Channel Model

In case of VLC, the channel can be modeled similarly by:

(3) $$y=hx+n_{\mathrm{VLC}},$$

where now *h* is the VLC channel gain, modeling the impulse response, and $n_{\mathrm{VLC}}$ are noise samples with real values with normal distribution $N(0,\sigma_{V}^{2})$ for a noise variance $\sigma_{V}^{2}$.

Thus, while for the DF case, the output is given by Equation (3), for the AF case, we have the output with noise contributions from both channels, according to Equation (4).

(4) $$y=hx+n_{\mathrm{PLC}}+n_{\mathrm{VLC}}.$$

As can be seen from Equations (3) and (4), the main difference is that in the AF case the PLC noise is carried with the signal of interest, but in the DF case this noise is eliminated once the signal is digitized. This will directly impact the signal-to-noise ratio (SNR) of the signal, which in turn impacts communication performance. One way to evaluate this is through a bit error ratio (BER) comparison between the two cases for an equal signal power.

**Figure 4:**
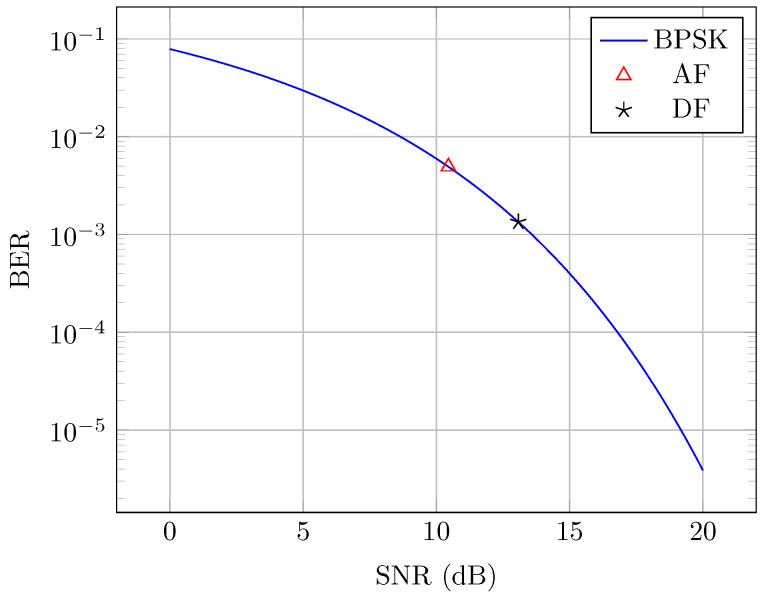
Comparison of the AF and DF case for the BPSK BER curve.

In Figure 4, this is presented considering a typical BER curve for the binary phase shift keying (BPSK) modulation used in digital communications and also as the main modulation scheme for this prototype [19]. The BPSK BER curve is governed by

(5) $${\mathrm{BER}}_{\mathrm{BPSK}}=\frac{1}{2}\mathrm{erfc}\left(\sqrt{\frac{E_{b}}{N_{o}}}\right),$$

where erfc() represents the error function, with a given argument corresponding to the bit energy $E_{b}$ and the power spectral density of noise $N_{o}$.

Projected in the curve are two main points of SNR vs. BER considering a signal with same power and subjected to both noise models as showed by Equations (3) and (4).

As can be observed, the DF case shows better performance with an SNR around 13.1 dB and thus a BER around 1.34 × $10^{-3}$, while the AF case has an SNR of around 10.5 dB and a BER of around 4.9 × $10^{-3}$. The reduction in the SNR, and consequently the increase in BER in the AF case is due to the presence of PLC noise, implying that this component would have been cared out along the PLC-VLC system up to the LED current. However, for the DF case, digital signal processing techniques and nonlinear processing may be applied in order to drastically eliminate such noise component; therefore, this shows an increase in the quality of the signal through and increase in the SNR and decrease in BER [20].

Thus, because of these two main addressed issues, the DF solution is chosen as a more interesting way of integration. It aims to digitize the signal through an analog-to-digital converter, process it with the help of a digital processor, and then send this digital signal to the LED load controller.

## 4. System Design and Implementation

The premises for the development of the system must align with what is applied in terms of LED-based lighting engineering. Guidelines are also established for VLC applications, especially regarding the data transmission rate. In this regard, a rate favorable for PLC applications under the utilization of the PRIME protocol is sought. PRIME is chosen as the main application of PLC due to its popularity and usage in the electric power supply environment. It is a PLC technology devised for bandwidth of up to 500 kHz and data rates from 5 kbps up to 1 Mbps, depending on the target application [21].

**Figure 5:**
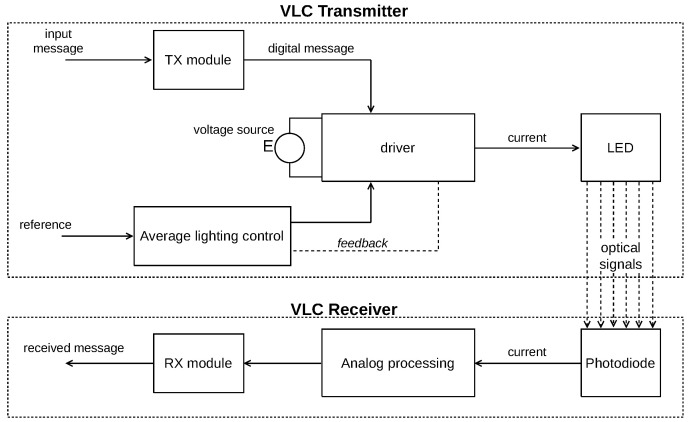
VLC system block diagram architecture.

Figure 5 displays a block diagram of a typical VLC system with average current regulation control. In this setup, the input message is a result of a successful transmission of the PLC system connected. The PLC Board #2 in Figure 1 forwards the processed message, which will be delivered by a VLC TX module that will send this digital signal to the LED driver in the form of a given modulation scheme. The dual purpose LED driver not only controls the average current level for lighting, but also modulates the instantaneous waveform of current for communication purposes. The rest of the system follows the same logic already mentioned in latter sections.

Hence, through a power electronics perspective, in [22,23,24], the authors provide a synthesis of the behavior of dual-function controllers regarding data rate and efficiency. According to this study, the general conclusion obtained coincides with the assertion that switched converters, although efficient, lack transmission capacity, while linear converters, although capable of processing high transmission rates, lack efficiency compatible with lighting applications.

Therefore, departing from these conclusions on the VLC literature, a given scenario is proposed to devise the PLC-VLC system under a series of premises that would rely on a prototype to be applied in the lighting industry. The main points of development of the project for the electronic circuit in question are presented as follows:

- The 48 V DC input bus: At this point, the standard 48 V bus becomes prominent in DC network research for processing electrical power intended for lighting. At this voltage level, applications for lighting and telecommunications for networks in commercial and residential applications are mainly assigned [13]. Additionally, it favors the use of power factor correction elements, as intermediaries between a 50/60 Hz network (PLC channel) and the lighting application based on the 48 V bus.
- Continuous conduction mode operation: Although it requires larger volume and higher inductance coils, the use of DC-DC converters operating in continuous conduction mode (CCM) is attractive for VLC. Firstly, the use of the converter in CCM allows for greater attenuation of switching harmonics compared to discontinuous conduction mode (DCM) for a constant peak current. Additionally, starting from fundamental converters, such as buck, boost, and buck–boost, operation in CCM allows for good power regulation regardless of load properties. Thus, CCM mode is applied to implement these two characteristics of interest: higher filtering capacity of switching harmonics and greater controllability of output power using the converter’s duty cycle [25].
- Single power control stage: Power control delivered to the load is achieved through the action of a single semiconductor switch. This premise reduces the number of passive elements in the electronic circuit, as well as active processing elements to understand the use of converters used in the lighting industry. Therefore, the use of a derived secondary bus is chosen, which will be analyzed below.

### 4.1. Converter Proposal

In [24], the use of linear amplifiers surrounded by design constraints is applied in order to deliver a high efficiency prototype with high bandwidth. This idea is considered an inspiration for the prototype of the VLC LED driver proposed in this paper.

**Figure 6:**
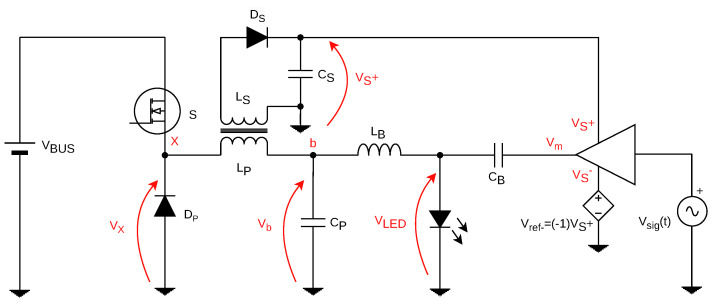
Proposed hybrid converter topology.

Figure 6 shows the base topology proposed. A buck converter is used to process power from a voltage source in order to deliver both functions of average lighting and VLC transmission. A secondary winding in the form of $L_{S}$ is provided to deliver a voltage that powers a linear power amplifier (LPA). Here, the LPA is seen as a secondary load that will be powered by the secondary winding of the converter. Moreover, a symmetrically-fed LPA is proposed in order to avoid distortion and allow for a zero DC input signal to be used as a the main VLC signal for communications. A voltage-dependent voltage source is therefore added in order to provide the negative voltage level $V_{\mathrm{ref}-}$ for the amplifier, based on a multiplicative factor of −1 of the voltage delivered by the $L_{S}$ winding (node $V_{S+}$). Hence, this symmetrical voltage range opens the door for the use of more robust commercial solutions of linear amplifiers, such as operational amplifiers which are less subjected to noise and have better input/output features for signal processing.

Furthermore, a bias-T circuit, consisting on the pair $L_{B}$ and $C_{B}$, is proposed to serve as a coupling system to deliver at the output a DC level of current, biasing the LED load, and also an AC signal corresponding to the VLC signal with a given modulation coming from node $V_{m}$.

This choice topology is based on a significant criterion that has already been mentioned regarding the proposal of the PLC-VLC system. Not only should this electronic circuit be capable of processing the communication signals of the two systems, but it should also correspond to the state-of-the-art criteria regarding the quality and requirements of lighting systems. Thus, efficiency becomes a fundamental characteristic to be studied in depth, along with the electronic system’s ability to process signals in different frequency ranges for communication purposes.

### 4.2. Converter Waveforms

**Figure 7:**
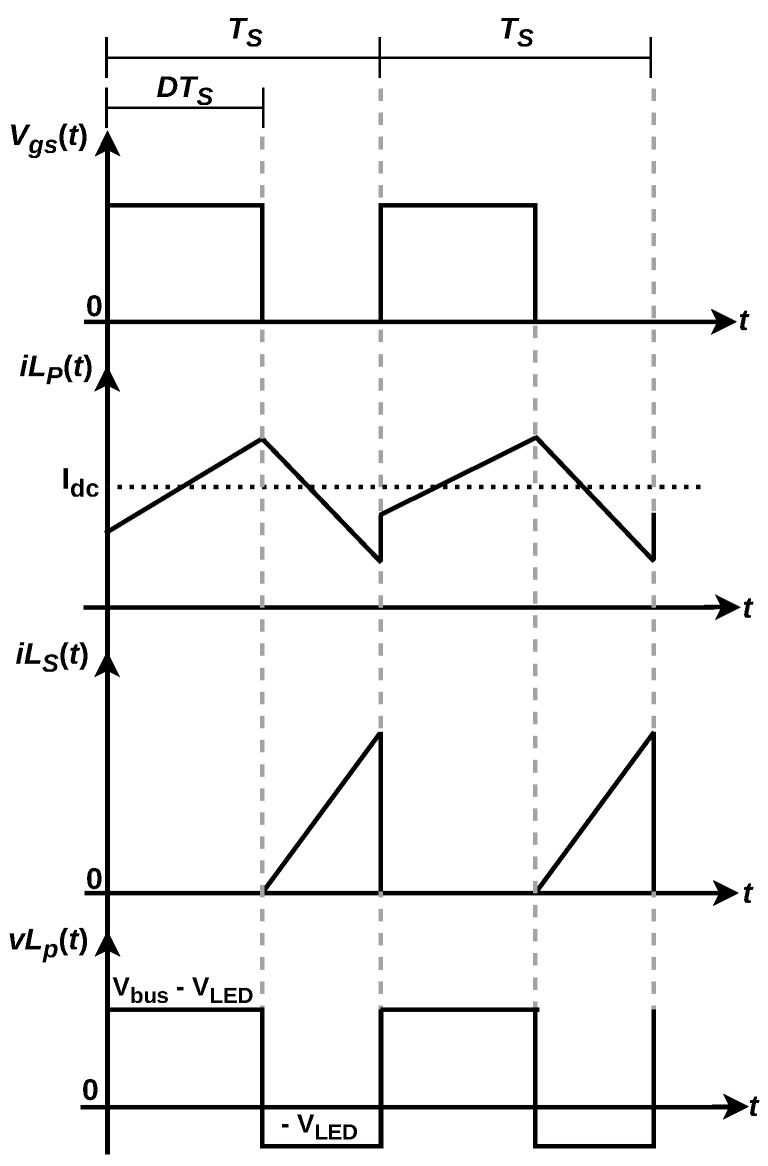
Waveforms on the coupled coil of the converter.

Figure 7 displays the operating steps, the current waveforms and the voltage behavior on the main coupled coil, responsible for powering both outputs. They are given in function of the command signal $v_{\mathrm{gs}}\left(t\right)$ that controls the on-off behavior of switch *S*. The polarity of the coupled coil pair ($L_{P}$,$L_{S}$) is devised in a way so the average output voltage at the LED load directly bias diode $D_{2}$ during the off stage, allowing for the transfer of power to the secondary. Following, $C_{S}$ holds the voltage to feed the LPA for VLC.

Energy stored in coil $L_{P}$ is transferred to both outputs during the discharge of $L_{P}$ (off stage of switch *S*). Hence, an asymmetrical charge/discharge behavior is observed when the coil assumes the loading of the secondary along with the primary. The average voltage on the secondary is a weighted value of the average voltage $V_{\mathrm{LED}}$, making it a design variable dependent on the transformer ratio of the main coil. This voltage is addressed according to the amount of power desired for the VLC function, which will be given in function of the current magnitude signal responsible for communications.

### 4.3. Frequency-Domain Modeling

In this section, we present the mathematical formulation driven based on the proposed converter. The goal is to provide a model in order to present the dynamics imposed by the use of such converter to the communication signal that will be sent via the LED emitted light.

First of all, the topology has two clear objectives: (i) to supply power for communication and lighting and (ii) to add the two corresponding signals to the output of the LED load without affecting the frequency response of the VLC signal. Hence, these tasks are translated into delivering with good quality two major current components to the output: A DC component for lighting and an alternating component corresponding to the message signal, with a given bandwidth and modulation technique.

Additionally, by observing Figure 6, in order to add these two characteristics, the pair $C_{B}$ and $L_{B}$ form a diplexer circuit that couples the DC signal responsible for lighting along with the VLC signal into the output current. Moreover, the dynamics of the converter’s output, to regulate the average level of lighting, must also be inserted into our mathematical formulation, in the form of the coil and capacitor pair $L_{P}$ and $C_{P}$, respectively.

**Figure 8:**
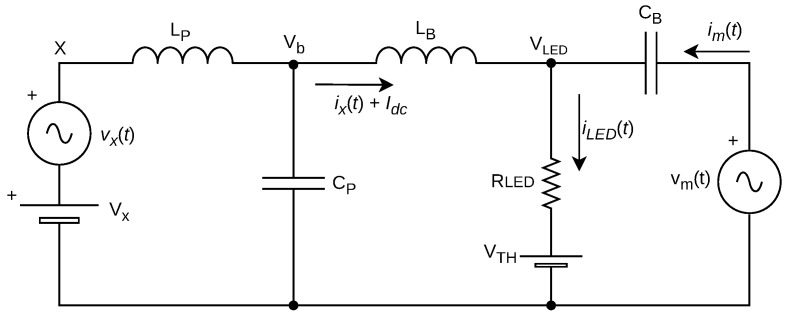
Circuit model of the dynamics of proposed converter.

Thus, a circuit model can be extracted from the proposed converter. Here, the LED is depicted by its well-consolidated linear model composed of a resistance and a voltage threshold consisted of a voltage source [26]. As displayed in Figure 8, it represents the dynamics of the passive components of the converter into the frequency response of the LED current. Note that in this assumption, the LED is effectively biased, thus eliminating the ideal diode present in the load’s mathematical model.

Furthermore, source $v_{m}\left(t\right)$ represents the output signal of the linear amplifier, hence the message signal, being treated here as an ideal voltage source. On the left-hand side of Figure 6, the diode/transistor pair generates a PWM signal composed of a DC level $V_{X}$, responsible for the average lighting, and an interference component $v_{x}\left(t\right)$ corresponding to the PWM harmonics. Thus, the model represents the PWM influence as a DC voltage source and an alternating source of interference. Finally, the noise component is then added to the $i_{LED}\left(t\right)$ as a total stochastic component representing all sources of noise during transference.

Therefore, the LED load current can be decomposed into four components, as follows:

(6) $$i_{\mathrm{LED}}\left(t\right)=I_{\mathrm{dc}}+i_{m}\left(t\right)+i_{x}\left(t\right)+i_{n}\left(t\right),$$

where $I_{\mathrm{dc}}$ represents the contribution of the average value of current, responsible for illumination; $i_{m}\left(t\right)$ is the component of the VLC message signal, responsible for sending information; $i_{x}\left(t\right)$ is a deterministic interference component, due to the frequency components of the PWM signal of the converter; and finally $i_{n}\left(t\right)$ is a stochastic noise component, and it depends on the characteristics of the channels involved in the flow of information.

Additionally, all current components mentioned must be considered while designing an electronic circuit to transmit information, while $I_{\mathrm{dc}}$, $i_{m}\left(t\right)$, and $i_{x}\left(t\right)$ can be addressed via circuit design, $i_{n}\left(t\right)$ is dependent on channel characteristics, and is directly affected by either an AF or DF solution.

Moving forward, by applying the Laplace Transform in Equation (6), it is possible to analyze the frequency response of the signal that will be transmitted via light to the optical channel by transforming the output current:

(7) $$I_{\mathrm{LED}}\left(s\right)=I_{\mathrm{dc}}\delta \left(s\right)+I_{m}\left(s\right)+I_{x}\left(s\right)+I_{N}\left(s\right),$$

where the DC component is manifested in the *s* domain through a Dirac delta function δ(s), and the noise component $I_{N}\left(s\right)$ is composed of its own response in the frequency domain. In addition, the other components $I_{m}\left(s\right)$ and $I_{x}\left(s\right)$ can be represented by their respective input stimuli, considering the dynamics of the transfer function of each with respect to the elements of the circuit model in Figure 8.

Thus, we have the following equation:

(8) $$I_{\mathrm{LED}(s)}=I_{\mathrm{dc}}\delta \left(s\right)+V_{m}\left(s\right)H_{m}\left(s\right)+V_{x}\left(s\right)H_{x}\left(s\right)+I_{N}\left(s\right),$$

where $V_{m}\left(s\right)$ and $V_{x}\left(s\right)$ represent the Laplace Transform of the voltage signals $v_{m}\left(t\right)$ and $v_{x}\left(t\right)$, respectively, and the functions in the *s* domain $H_{m}\left(s\right)$ and $H_{x}\left(s\right)$ represent the circuit dynamics when viewed by each of the independent voltage sources. Therefore, the problem becomes one of signal superposition, where the analysis of the inputs can be performed separately, disregarding all others.

Thus, by applying the Laplace Transform to the circuit and performing an analysis of Kirchhoff’s currents and voltages, we expected a fourth order circuit with four poles in its transfer function. We effectively arrive at the equations that govern the dynamic behavior of the circuit with respect to the mentioned voltage inputs, presented in the following equations:

(9) $$\begin{array}{cc} H_{x}\left(s\right) & =\frac{1}{A+sB+s^{2}C+s^{3}D+s^{4}E},\;\\ H_{m}\left(s\right) & =\frac{K}{A+sB+s^{2}C+s^{3}D+s^{4}E},\;\mathrm{where}\\ A & =R_{\mathrm{LED}},\;B=L_{B}+L_{P},\;C=C_{P}L_{B}R_{\mathrm{LED}}+C_{B}L_{B}R_{\mathrm{LED}}+C_{P}L_{B}R_{\mathrm{LED}},\\ D & =C_{B}L_{P}L_{B},\;E=R_{\mathrm{LED}}C_{B}C_{P}L_{P}L_{B}\;\mathrm{and}\\ K & =C_{B}\left(C_{P}L_{B}L_{P}s^{2}+L_{B}\right). \end{array}$$

Moreover, the electric current is transformed as a light signal by the current-to-light transformation performed by the LED load. Thus, on the optical receiver side, while the signal is being transmitted via the optical channel, the channel model follows an impulse response approach, described by the following equation and its Laplace Transform pair [19]:

(10) $$\begin{array}{cc} r(t) & =\epsilon i_{\mathrm{LED}}\left(t\right)⊛h_{\mathrm{VLC}}\left(t\right),\;\\ R(s) & =\epsilon I_{\mathrm{LED}}\left(s\right)H_{\mathrm{VLC}}\left(s\right), \end{array}$$

where r(t) is the received signal on the photodiode, ϵ denotes the receiver’s photodiode responsivity, given in A/W, and ⊛ denotes the convolution operator between the LED current and the VLC channel impulse response $h_{\mathrm{VLC}}\left(t\right)$, which is manifested as a multiplication between the corresponding frequency responses in the Laplace domain.

Finally, this mathematical formulation is used to simulate the dynamics of the converter and its effect on the message signal being delivered. The next section presents the converter guidelines, equations, and reasoning, which will later be used along with the Laplace model to obtain the frequency responses of the proposed solution.

### 4.4. Converter Design Guidelines

In the guidelines, we show the design equations that will be used to design the passive elements of the converter. First, it is interesting for the converter part to filter the switching ripple generated by the PWM in order to decrease signal interference. Second, regarding the hybrid circuit, the coupling capacitor is particularly important for this project, which operates with fixed bandwidths for PLC, where these bandwidths overlap with the ranges of frequencies commonly used for converter switching.

#### 4.4.1. Converter’s Main Coil $L_{P}$

The main coil corresponds to the coil of the converter that processes the input power and delivers it to the VLC and lighting outputs. The coil follows the traditional design of a buck converter, as presented in the equation below [25]:

(11) $$L_{p}=\frac{\left(V_{\mathrm{bus}}-V_{\mathrm{LED}}\right)V_{\mathrm{LED}}}{\Delta I_{L}\cdot f_{s}},$$

where $V_{\mathrm{bus}}$ is the input bus voltage, $V_{\mathrm{LED}}$ is the output voltage across the LED load, $\Delta I_{L}$ is the current ripple in amperes, and $f_{s}$ is the switching frequency. It is also important to mention that $V_{\mathrm{LED}}=V_{\mathrm{th}}+R_{\mathrm{LED}}I_{LED}$, that is, the voltage based on the linear model of the load, where $I_{O}$ is the average value of the output current.

Moving forward, the secondary coil $L_{S}$, responsible for biasing the linear amplifier and thus delivering power to the VLC function, is designed according to the transformer ratio between secondary and primary $L_{P}$, given as follows:

(12) $$L_{S}/L_{P}={\left(\frac{N_{S}}{N_{P}}\right)}^{2},$$

where $N_{S}$ is the number of turn for the secondary coil and $N_{P}$ is the number of turns for the primary coil.

#### 4.4.2. Design of the Amplifier’s Voltage Bus

The secondary voltage bus is responsible for regulating the amount of power to be delivered to the linear element for processing VLC.

The amplitude of the VLC current signal obeys the transfer equation that governs the output voltage signal of the linear element to the LED load current:

(13) $$|V_{m}\left(s\right)|=|I_{m}\left(s\right)||H_{m}\left(s\right)|,$$

where $|I_{m}\left(s\right)|$ is a design magnitude of the current as a function of the permissible bandwidth for VLC and $H_{m}\left(s\right)$ is the transfer function of the diplexer circuit in Equation (9).

A maximum amplitude excursion of the linear element at its output, called $V_{m}$, is assumed. The peak-to-peak value, therefore, is $2V_{m}$. As the secondary coil is coupled to the average value of the output of the hybrid converter, the transformation ratio is employed as follows:

(14) $$\frac{V_{s}}{2V_{m}}=\frac{V_{\mathrm{LED}}N_{s}}{N_{p}},$$

where $V_{\mathrm{LED}}$ denotes the average voltage at the output of the LED regulated by the buck converter circuit.

Since the secondary voltage is effectively determined by the transformation ratio, we express it as a function of the current magnitude by replacing the magnitude of $|V_{m}\left(s\right)|$ into $V_{s}$:

(15) $$\frac{N_{s}}{N_{p}}=\frac{2|I_{m}\left(s\right)||H_{m}\left(s\right)|}{V_{\mathrm{LED}}}.$$

Therefore, Equation (15) relates the design elements of the DC-DC converter to the voltage required at the secondary to process a certain current amplitude. It makes a bridge between the power requirements for the proposed communication system, addressing the available bandwidth under the knowledge of the converter dynamics.

#### 4.4.3. Converter’s Output Capacitor $C_{P}$

Therefore, the output capacitor is calculated based on a factor $k_{c}$ that dictates its reactance depending on the impedance of the LED load itself, given by:

(16) $$C_{P}=\frac{k_{c}}{2F_{\mathrm{VLC}}R_{\mathrm{LED}}}.$$

Hence, if $k_{c}>0$ and $F_{\mathrm{VLC}}$ are the fundamental frequencies of the information carrier signal, then the capacitor is given by its reactance proportional to the impedance of the LED load at the frequency of interest.

#### 4.4.4. Amplifier’s Coupling Capacitor $C_{B}$ (Diplexer’s Capacitor)

Capacitor $C_{B}$ forms an important element that couples the alternating communication signal to the output current. Although its design may be inferred by a more complex use of the converter’s transfer function as mentioned, some pre-assumptions can be inferred in order to simply its impact on the coupling of the information signal to the LED current.

To design the capacitor, we start from a simplified version of the output circuit, where it is assumed that the inductance of the diplexer coil $L_{B}$ is large enough to block any values of alternating signal from the output current of the linear amplifier LT1210, from Linear Technologies, Milpitas, CA, USA.

**Figure 9:**
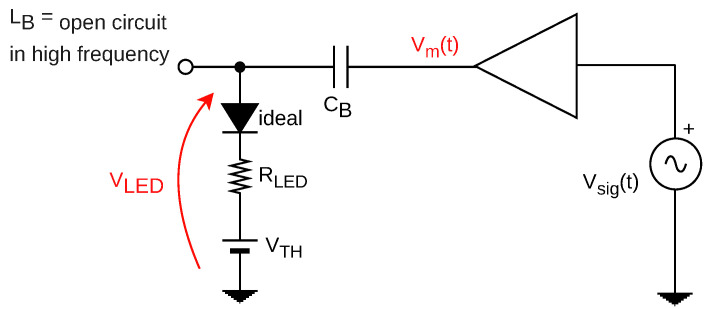
Simplified first order circuit to determine coupling capacitor.

Thus, the diplexer circuit is simplified to an RC first order low-pass circuit, as illustrated in Figure 9.

Therefore, using an approach of the circuit in Figure 9 in its AC model, the capacitor $C_{B}$ forms a high-pass filter with the resistance of the LED load. Therefore, it can be determined according to its cutoff frequency $F_{C}$ through the equation:

(17) $$F_{C}=\frac{1}{2R_{\mathrm{LED}}C_{B}},$$

By putting $C_{B}$ into evidence, where $F_{C}$ must match the communication requirements of bandwidth and pulse shape being used to transmit the VLC signal to the LED current.

#### 4.4.5. Diplexer’s Coil $L_{B}$

The diplexer coil serves as a high-impedance mechanism for the alternating part of the current. When looking at the perspective of the current it needs to block, its value is based on the desired reactance of the coil within the range of the VLC signal. Thus, it is desirable to put it in terms of the resistance of the LED load, under a proportionality factor $k_{f}$:

(18) $$L_{B}=\frac{R_{\mathrm{LED}}k_{f}}{2F_{\mathrm{VLC}}}.$$

Following the same approach for $C_{B}$, it is desirable that $L_{B}$ possesses a much higher impedance when compared to the LED load path. Thus, if $k_{f}>0$ and $F_{\mathrm{VLC}}$ are the fundamental frequencies of the transmission carrier, the reactance at the signal frequency is greater than the impedance of the LED load.

## 5. Methodology

Based on the arguments provided in the former sections, the experimental setup proposed is designed considering the following assumptions:

- Data-rate target: A dual-purpose PLC-VLC system capable of operating under an overall data rate of 100 kpbs for the VLC part, devised for low to moderate data rate applications. The choice of 100 kbps allows for the use of readily available, cost-effective microcontrollers and digital signal processing (DSP) units without necessitating high-performance, high-cost components;
- Modulation scheme for VLC: The setup comprehends a BPSK modulation scheme with rectangular pulse shape that has been already proposed as a standard technique in the literature [27]. BPSK can be implemented with basic, low-cost hardware components, which is critical for a dual-purpose LED driver that functions both as a VLC transmitter and a lighting source. Therefore, the hardware requirements for BPSK are minimal compared to more complex modulation schemes like Quadrature Amplitude Modulation (QAM) or Orthogonal Frequency Division Multiplexing (OFDM);
- Lighting requirements: An LED load of up to 800 lm and 10 W, devised using regular surface mount (SMD) LEDs and with enough heat sinking to avoid heat-oriented distortions on the light. A standard light output for many household applications is 800 lm, equivalent to the light produced by a traditional 60 W incandescent bulb. This makes it a suitable and familiar choice for end-users [28].

### 5.1. Hardware Components

**Figure 10:**
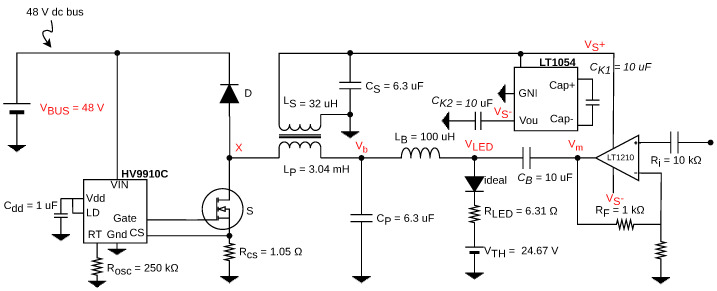
Simplified first order circuit to determine coupling capacitor.

Figure 10 displays the converter prototype devised based on the schematic, along with the values of the passive components. The main difference with respect to the schematic of Figure 6 is the change in the reference node, which is demanded by the use of the controller chip HV9910C, from Microchip, Chandler, AZ, USA.

The switching frequency of the transistor of Figure 10 was configured to be at 100 kHz in order to accommodate the coil’s size in a fitted prototype. Of course, this choice will also impact the filtering elements that will have to attenuate the PWM components at the output in order to mitigate interference on the communication signal.

The passive components were calculated according to the design guidelines mentioned above. Table 1 presents a list of electronic components used in the prototype. The values of the passive components are described. Here, the LED is displayed by its linear model, which will be explained next.

### 5.2. Design of LED Load

**Figure 11:**
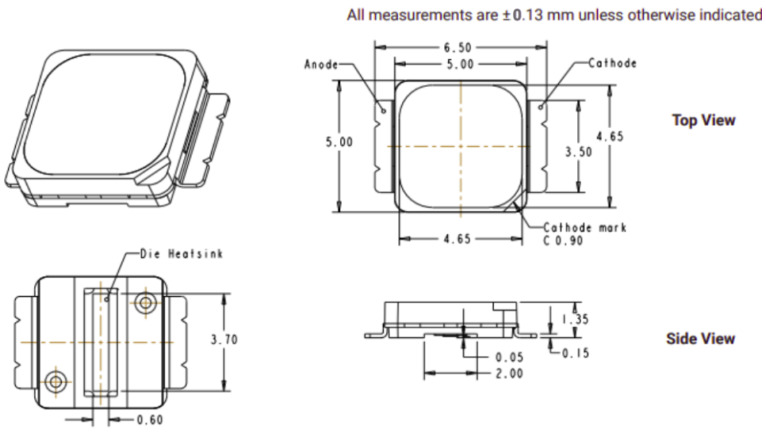
Dimensions of SMD LED for the load.

The design of the converter is based on a given LED load, here represented by its equivalent linear model, consisting of a resistance in series with an ideal diode and a voltage source. The load was designed to represent a 800 lm light source, with a nominal current of 340 mA and nominal power of 10 W. The load uses 9 surface mount LEDs of part number MX6AWT-AI-R250 XLAMP 5000K, in which the dimensions are depicted in Figure 11.

Thus, the following characteristics can be highlighted for the LED load:

- Maximum power of 10 W;
- Average luminous flux of 800 lm, equivalent to 350 lux at a distance of 1 m and a beam angle of 55 degrees, corresponding to a commercial bulb-type lamp application;
- Nominal average current of 350 mA;
- General operating voltage of 28.65 V.

In addition, to clarify the limits of this work, the LED load model does not consider the LED frequency response, thus assuming the load to have enough bandwidth for the targeted VLC application. Moreover, the load is designed without considering heat transfer dynamics and the impact on the LED light flux generation, hence providing an oversized heat sink.

**Figure 12:**
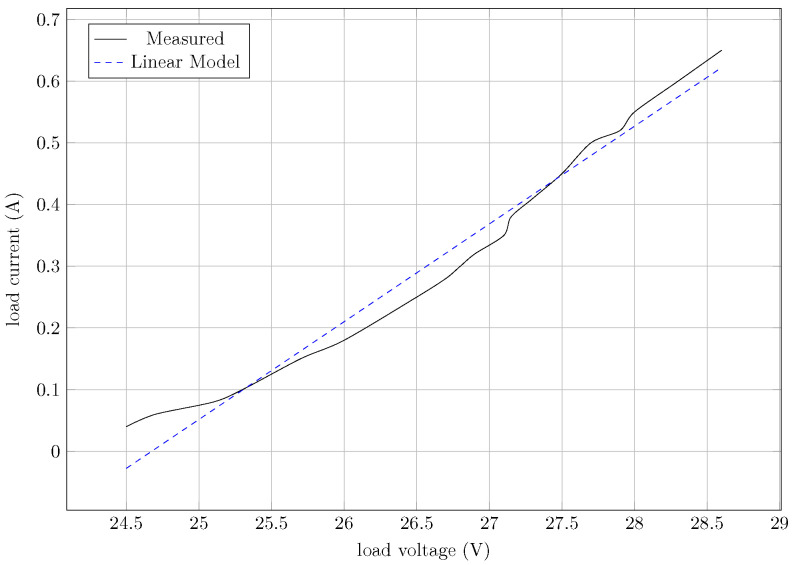
Linear model approximation of IxV curve of the proposed LED load.

Figure 12 shows the experimental IxV curve and its linear approximation used as the electric model. The analysis of the slope and interception of the linear model provides the values of $r_{\mathrm{LED}}=6.31$ Ω and $V_{\mathrm{TH}}=24.67$ V.

### 5.3. Communication Hardware

The PLC network described in this study is implemented using evaluation boards manufactured by Microchip, specifically the PL360G55CF-EK evaluation boards. These boards feature the following key components:

- Microcontroller (MCU): The evaluation boards are equipped with a microcontroller from the SAM55 series. The MCU serves as the central processing unit responsible for executing the firmware and managing data transmission and reception;
- PLC Modem (PL360): Each evaluation board integrates a PLC modem, specifically the PL360 model. The PLC modem facilitates communication over the power lines by modulating digital signals into analog waveforms suitable for transmission over the power grid and demodulating received signals back into digital data. The modem also automatically follows the PRIME standards specifications;
- Terminal Inputs: The evaluation boards support communication with external devices, such as desktop PCs and other hardware, through terminal inputs. These inputs allow for the transmission of messages and commands to the PLC network, enabling control and data exchange with the connected devices.

The UART module on board #2 serves as the interface through which commands, instructions, and data are transmitted to the LED driver. By establishing a UART communication link between the evaluation boards and the LED driver, the BPSK modulation scheme is performed digitally, with the operating system of the MCU controlling the bit stream at 100 kbps.

**Figure 13:**
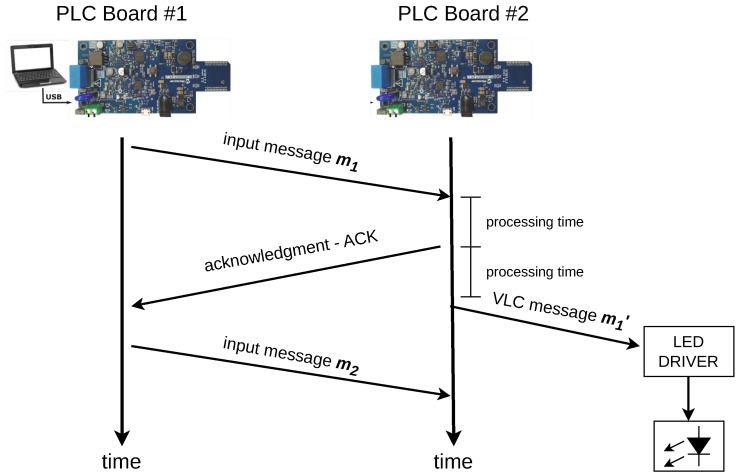
Time diagram of the PLC network and interaction with the VLC system.

Finally, Figure 13 displays a time diagram of the PLC communication networks and its interaction with the PLC-VLC setup. A message $m_{1}$ is sent by board #1 and forwarded by board #2 to the VLC setup. The board processes and copies the content as a message $m_{1}^{\prime }$ and forwards to the LED driver in the form of a BPSK signal by using the dedicated UART module of the SAM55 MCU.

### 5.4. Simulation of LED Current

**Figure 14:**
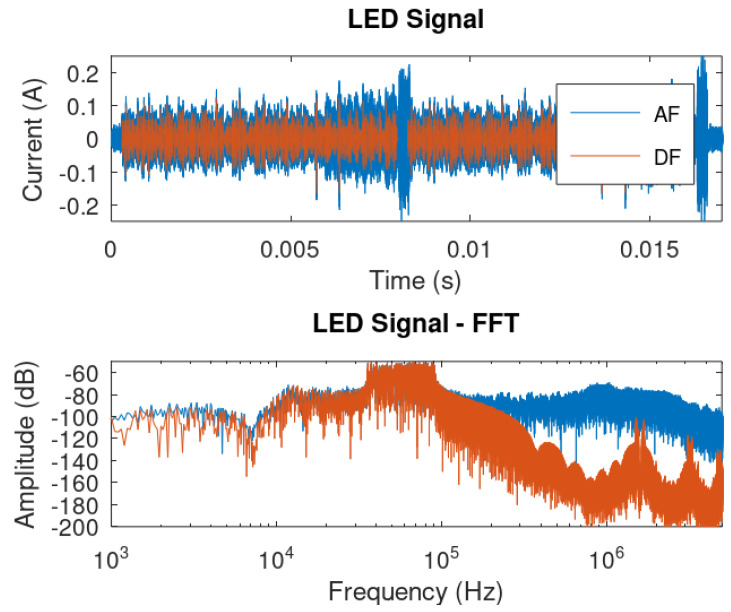
Waveforms in time and frequency domains of the LED current.

In this section, we provide a simulation considering a communication symbol of both AF and DF techniques. Figure 14 shows a waveform of the output LED current, along with their frequency spectra.

It is possible to observe the change in amplitude in order to comply with the same magnitude of energy displayed in the FFT spectra. That is, to sustain the same level of signal power, scattered across the bandwidth, the AF case requires more overall amplitude due to the bandwidth spectral leak that can be observe on the frequency components above 100 kHz.

Therefore, this observation also sustains our claim to use the DF case, as it is more efficient in terms of bandwidth efficiency and also can rely on off-the-shelf digital components that are of friendly use and can comply with the most used modulation schemes for both PLC and VLC.

### 5.5. VLC Receiver Design

**Figure 15:**
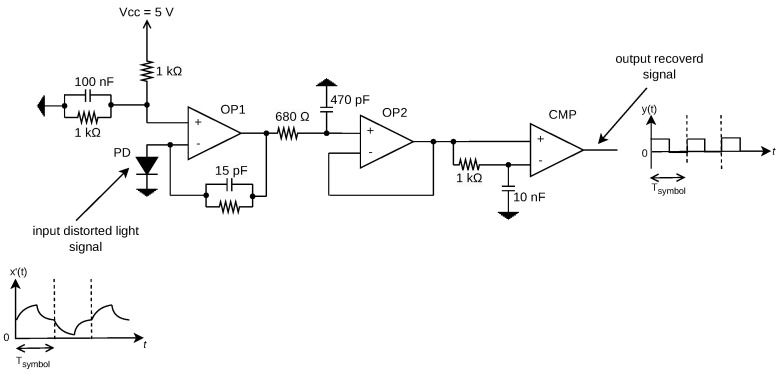
Schematic of receiver circuit.

The optical receiver is designed to detect a pulsed light signal with main frequency of 100 kHz (proportional to 100 kbps) corresponding to the BPSK signal with rectangular pulse shape. The circuit works as comparator between the bipolar waveform and its average value that recovers the initial bit stream that originated the BPSK signal, thus regenerating it and eliminating channel distortions under a certain SNR limit. Figure 15 shows the receiver circuit schematic, and Table 2 shows the main components. The circuit consists of three main stages, as follows:

- Transimpedance Stage (OP1): A transimpedance amplifier (TIA) is employed to convert the current signal generated by the photodiode when capturing light signals into a voltage signal, represented by the OP1 stage.
- Buffering stage (OP2): The buffering stage in OP2 is utilized to match impedances and acts as a voltage follower to mitigate high-frequency noise originating from the preceding stage.
- Comparison stage (CMP): The comparator stage, through comparing the amplified pulsed signal with its average value, proportional to the average light generated by the driver, reconstructs the signal waveform into a clean rectangular pulse waveform. This corresponds to the restored Binary Phase Shift Keying (BPSK) signal transmitted by the driver and it is ready for decoding by another digital processing unit.

### 5.6. Simulation of Frequency Response

**Figure 16:**
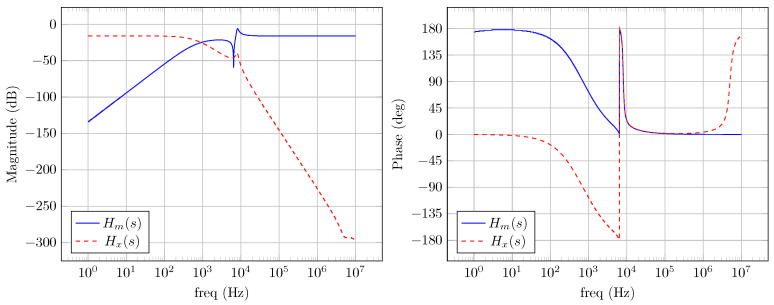
Frequency responses of the proposed prototype.

A simulation was performed in LTSpice considering the circuit model proposed in Figure 8. The goal is to check the converter’s frequency responses of the transfer functions that affect both the communication signal $H_{m}\left(s\right)$ and the PWM signal of the converter $H_{x}\left(s\right)$, the latter being treated as an interference. Thus, Figure 16 displays the simulated transfer functions frequency responses.

It is possible to observe that the system presents a flat response and zero phase at the bandwidth of interest of the communication signal. For the interference caused by the PWM, the system acts as a low pass filter with a starting attenuation of approximately −50 dB at the frequency of 10 kHz; therefore, it fulfills the objective of the diplexer circuit of coupling both communication signal with only the average value processed by the converter.

In conclusion, these preliminary results on the frequency response indicate a balanced design of the converter, making these values able to be tested in a prototype that will be described next.

## 6. Experimental Results and Discussion

This section will present the prototype designed following the theory described in the rest of the paper. To summarize our findings and objectives, the prototype describes a hybrid buck converter working as a dual-purpose LED driver for VLC and lighting. The VLC part works to receive a digital BPSK waveform generated by the PLC part of the system once it decodes the PLC message sent through the grid. Therefore, the converter reproduces the same BPSK signal in the output current of the LED load.

**Figure 17:**
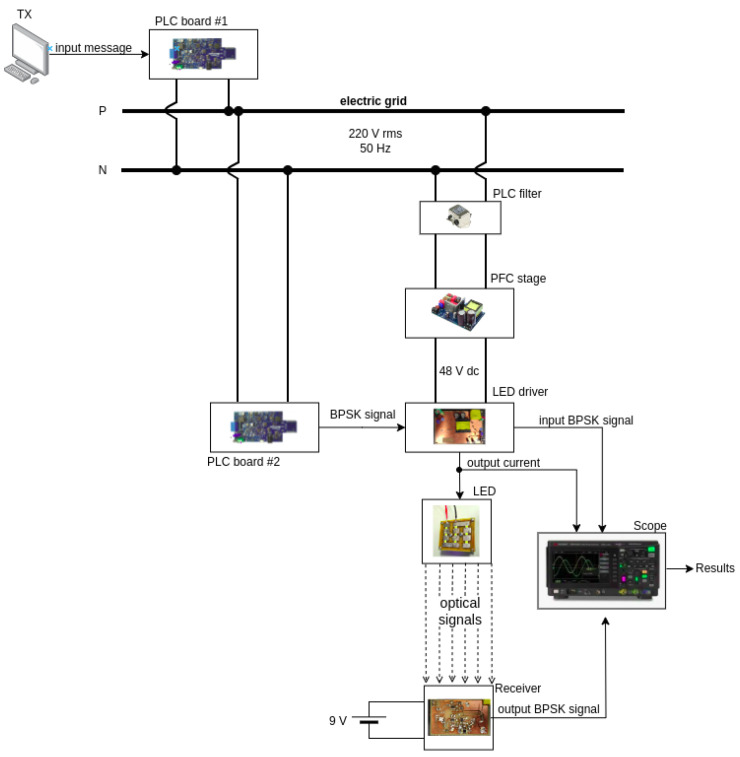
Block diagram of experiment.

Figure 17 displays a block diagram of the proposed setup. The electrical grid consists of a 60 Hz 220 V rms bus that is used as the communication medium for PLC and to power a PFC stage that converts that processes a 48 V DC bus for powering the LED driver. The board used to execute this task is a evaluation board designed by ST Microelectronics, part number EVAL-PSR01B-35W. A PLC filter coil (part number FN2010-10-06) is also used to isolate the interference from the PFC stage on the PLC network utilizing the grid. The receiver is powered by a 9 V battery, and thus isolated from the grid and separated by a distance of 1.2 m from the VLC transmitter, on a line of sight configuration.

**Figure 18:**
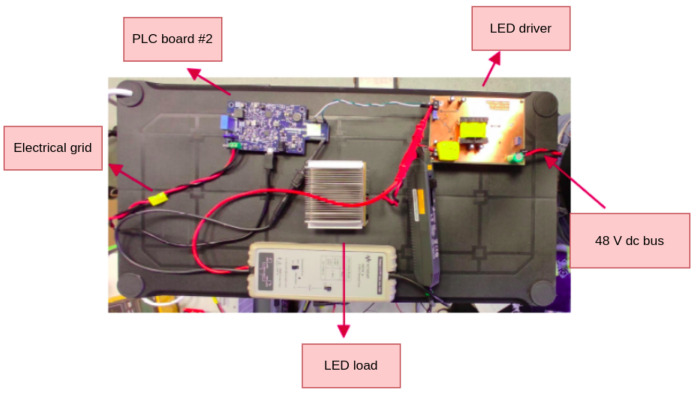
Top view of prototype.

**Figure 19:**
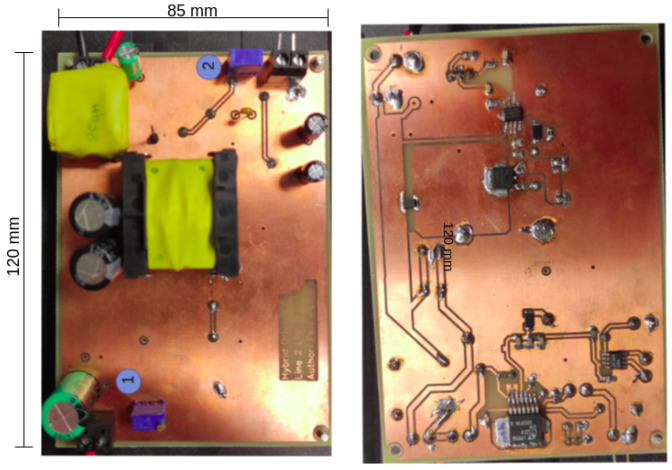
Receiver prototype.

**Figure 20:**
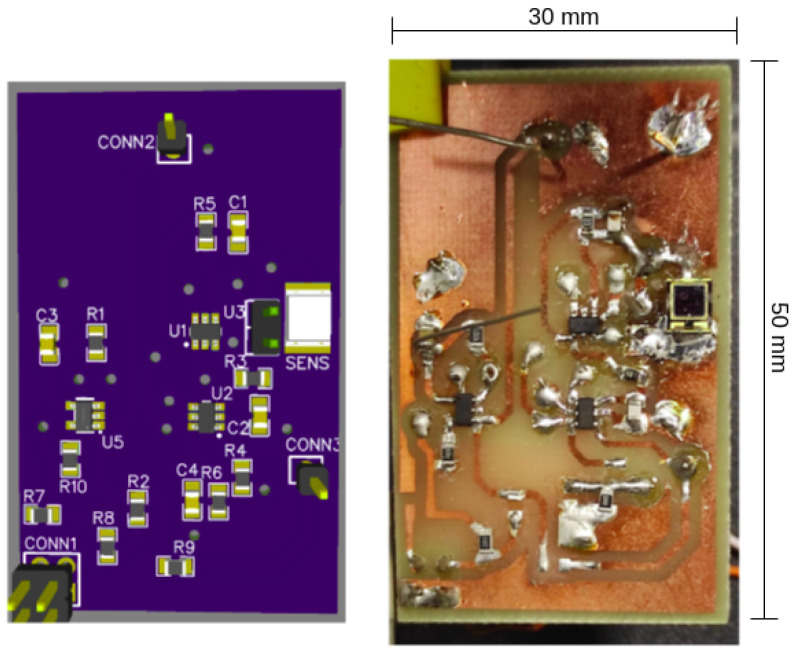
Receiver prototype.

Figure 18 displays a top view of the setup showing the Microchip board as the PLC board #2 connected to the driver. Figure 19 displays the designed PCB for the LED driver circuit, where (1) indicates the 48 V input and (2) indicates the input for the BPSK signal. Figure 20 displays the designed PCB for the receiver circuit.

**Figure 21:**
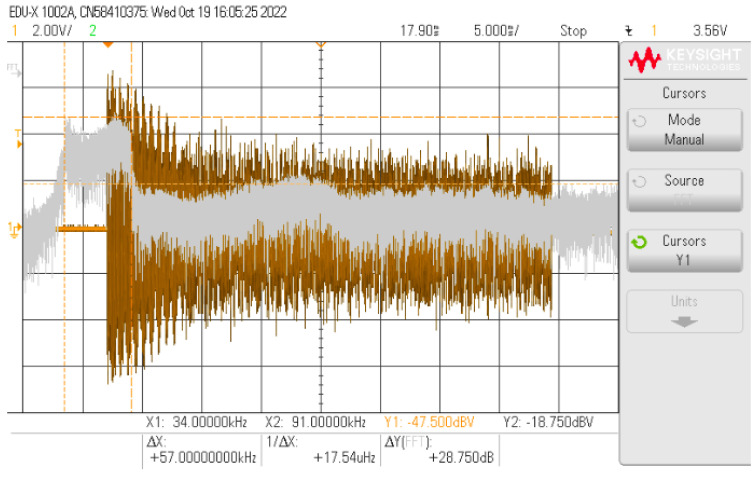
Spectrum of PLC message.

Figure 21 displays a message signal being sent over the PLC network. The signal consists of an modulated bandwidth corresponding to the CENELEC A recommendation, which is available in the PRIME protocol. The bandwidth occupies a total range of 57 kHz, from 34 to 91 kHz. This signal is captured by PLC board #2 and processed in order to create the BPSK signal for VLC.

**Figure 22:**
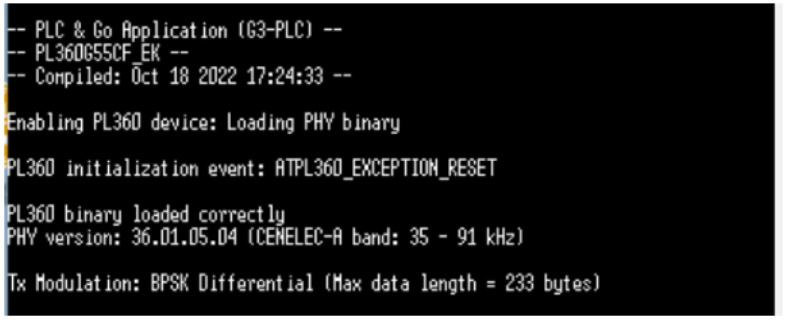
PLC parameters for modified PLC & Go application.

Figure 22 displays an input terminal message of the operating system devised for PLC board #2 that handles the message signal traffic between the PLC and VLC applications. This operating systems is a modified version of the open code PLC & Go application, devised by Microchip and modified to accommodate the needs for the PLC-VLC prototype.

**Figure 23:**
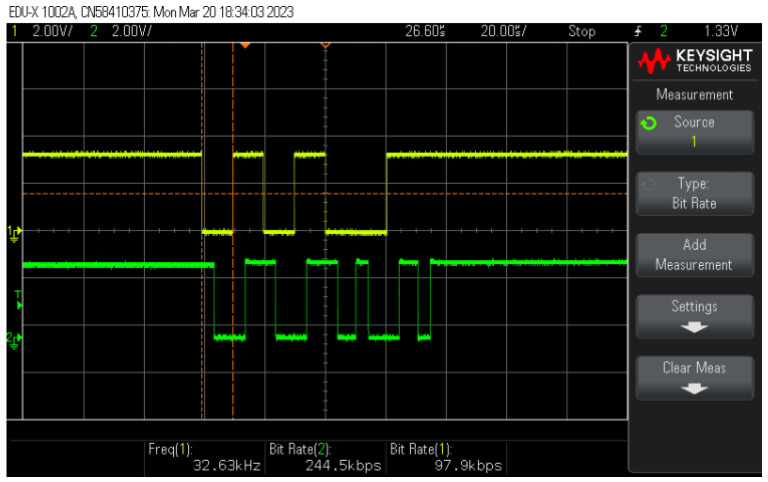
Bit stream (**top**) and its BPSK signal (**bottom**).

Figure 23 displays an example of the BPSK signal being used for communications and its bit stream counterpart. The logic is performed by the operating system inserted in PLC board #2. At each bit transition, the waveform shifts its phase by 180 degrees to identify the transition. The final bits on the BPSK signal also signal to the receiver the end of a successful transmission, and they are used as flags to identify when a message burst ends.

**Figure 24:**
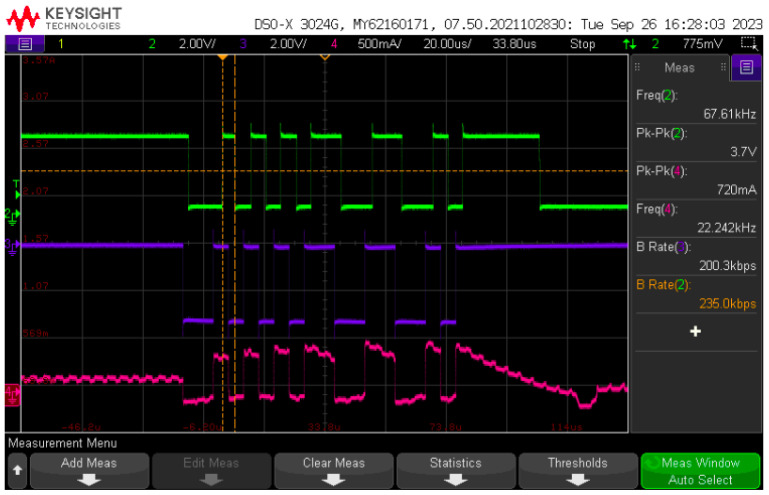
BPSK signal. From top to bottom: optical receiver output (green); input BPSK signal (blue); LED current (magenta).

Figure 24 displays a sample of the BPSK signal processed by PLC board #2 and forwarded to the LED current. It is possible to see the distortion caused by the rectangular pulse on the LED current due to the effect of the transfer function low frequency pole and resonance, as displayed in the frequency response of Figure 16.

**Figure 25:**
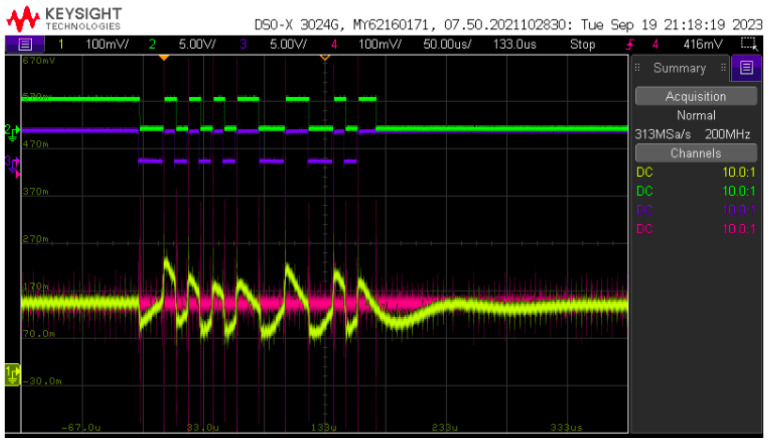
BPSK signal on the receiver. From top to bottom: input BPSK signal (green); optical receiver output (blue); received optical signal (yellow); average value reference for comparator (magenta).

Figure 25 displays the receiver waveforms and the inputs for the comparator circuit stage. The output signal from the photodiode suffers more distortion due to the low frequency response of the current-to-light conversion of the LED convoluted with the photodiode’s frequency response and the effect of the optical channel noise. However, even in this case, the nonlinear compensation given by the comparator stage of the receiver managed to regenerate the BPSK signal.

## 7. Conclusions

This study presents the design and implementation of a dual-purpose LED driver system that functions as both a lighting source and a Visible Light Communication (VLC) transmitter. The system integrates with a Powerline Communication (PLC) network under the PRIME G3 standard, facilitating the transmission of data through existing power lines. The key contributions and findings of this work are summarized as follows:

- The PLC network was developed using Microchip evaluation boards (part number PL360G55CF-EK), which incorporate a SAM55 series microcontroller (MCU) connected to a PL360 PLC modem. The system is programmed to send messages via terminal inputs from a desktop PC, demonstrating integration of PLC and VLC technologies;
- An experimental setup is proposed, with described guidelines to successfully reproduce both the VLC transmitter and receiver. The setup also explores the use of digital solutions and consolidated microprocessor units to overcome the challenges of integrating both systems;
- Guidelines and justification of the designed parameters are also explained and were used to develop a prototype in order to fulfill the requirements of a technology under the level 4 of the Technology Readiness Level guide, that is, a prototype validated under laboratory conditions;
- The system was validated through experimental results, including oscilloscope images that depict the signals traveling across the system. The comparison shows that, even in noisy and distorted conditions, the system could successfully reproduce the input signal message generated through extracting the information from the PLC network;
- The operating system responsible to demodulate and forward the PLC message can be found in the link provided in the Appendix A of this article.

In conclusion, this work provides a significant step towards the practical deployment of integrated PLC and VLC systems, highlighting their potential to transform modern communication networks through innovative use of existing resources.

## Figures and Tables

**Table 1:** Converter’s main components.

Component	Part Number
Converter’s MOSFET	IPD60R950C6ATMA1
Main and secondary diodes	PMEG10020AELPX
LPA	LT1210
Symetrical voltage converter	LT1054
Average Current Control	HV9910C
PLC Board for input signal	PL360G55CF-EK

**Table 2:** Receiver components.

Component	Part Number
OP1 and OP2	ADA4099
CMP—comparator	LT1716
Photodiode	TEMD5020X1

## Data Availability

Data are contained within the article.

## Appendix A

The operating system devised to handle PLC and VLC messages can be found in the following repository link: https:gitlab.com/felipe-loose/plc-and-go-rep (accessed on 18 September 2024).
